# Molecular and Clinical Predictors of Quality of Life in Chronic Rhinosinusitis with Nasal Polyps

**DOI:** 10.3390/jcm12041391

**Published:** 2023-02-09

**Authors:** Aina Brunet, Javier Milara, Soledad Frías, Julio Cortijo, Miguel Armengot

**Affiliations:** 1 Department of Otorhinolaryngology and Head and Neck Surgery, Hospital Universitari Bellvitge, 08907 Barcelona, Spain; 2 Institut d’Investigació Biomèdica (IDIBELL), 08908 Barcelona, Spain; 3 Department of Pharmacology, Faculty of Medicine, University of Valencia, 46010 Valencia, Spain; 4 Pharmacy Unit, University General Hospital Consortium of Valencia, 46014 Valencia, Spain; 5 Biomedical Research Networking Centre on Respiratory Diseases (CIBERES), Health Institute Carlos III, 28029 Madrid, Spain; 6 Department of Otorhinolaryngology, Hospital de Manises, 46940 Valencia, Spain; 7 Research and Teaching Unit, University General Hospital Consortium, 46014 Valencia, Spain; 8 Department of Otorhinolaryngology, La Fe University and Polytechnic Hospital, 46026 Valencia, Spain; 9 Molecular, Cellular and Genomic Biomedicine Group, Instituto de Investigación Sanitaria La Fe, 46020 Valencia, Spain; 10 Surgery Department, Faculty of Medicine, University of Valencia, 46010 Valencia, Spain

**Keywords:** interleukin-8, eosinophilia, quality of life, nasal polyp, corticosteroids

## Abstract

(1) Background: Factors influencing the quality of life (QoL) of patients with chronic rhinosinusitis with nasal polyposis (CRSwNP) are poorly understood. We set out to determine the predictive factors on patients’ QoL using the Sino-Nasal Outcome Test-22 (SNOT-22); (2) Methods: An ambispective analysis of data from patients diagnosed with CRSwNP in our institution. All the patients underwent a nasal polyp biopsy and completed the SNOT-22 questionnaire. Demographic and molecular data as well as the SNOT-22 scores were collected. Patients were classified in six subgroups considering the presence of asthma, non-steroidal drugs (NSAID) intolerance and corticosteroid resistance; (3) Results: The mean SNOT-22 score was 39. Considering the clinical parameters, the SNOT-22 value was significantly associated with NSAID intolerance (*p* = 0.04) and the endoscopic polyp score (*p* = 0.04). A high SNOT-22 value was also correlated with high tissue eosinophilia (*p* = 0.01) and high IL-8 expression; (4) Conclusions: Eosinophilia, IL-8 expression and NSAID intolerance can be used as predictors of worse QoL in patients with CRSwNP.

## 1. Introduction

Chronic rhinosinusitis (CRS) consists of a chronic inflammatory process of the nose and paranasal sinuses which affects 15% of the population worldwide. Precisely, it has been described that chronic rhinosinusitis with nasal polyps (CRSwNP) has a prevalence of 2–10.9% in western countries [1,2]. It is well known that CRS is a significant health problem and it has a negative impact on patients’ quality of life (QoL), besides causing economic implications due to the high cost of treatments [2,3,4].

There has been a rapid increase in the use of patient-reported outcome measures (PROMs) in studies of clinical effectiveness and quality of care [5]. PROMs are now an essential tool in the evaluation of care for chronic conditions such as CRS.

The severity of patients’ symptoms and their impact on health-related quality of life (QoL) can be measured using the validated QoL questionnaire, the Sino-Nasal Outcome Test 22 (SNOT-22). It creates a score out of 110 points that reflects the severity of symptoms. The SNOT-22 questionnaire is a PROM in sinonasal disorders [6]. It covers a range of problems including physical problems, functional limitations and emotional consequences. It reflects severity creating a score out of 110 points [2,6]. It is currently the most effective tool available for grading the severity and impact of clinical symptoms of CRS [2,5].

Surprisingly, there is a paucity of data in the published literature studying the role of clinical and molecular parameters in QoL using the SNOT-22. Our study sets out to determine the predictors of QoL in patients with CRSwNP, assessing different clinical and molecular parameters, in order to better understand this complex but common chronic disease.

## 2. Materials and Methods

### 2.1. Study Population

Adult participants (≥18 years) diagnosed with CRSwNP as per the European Position Paper in Nasal Polyps 2020 (EPOS 2020) with no clinical or endoscopic response to intranasal corticosteroids (INCS) for 3 months were prospectively recruited from a tertiary care center. The INCS used were budesonide, mometasone furoate, fluticasone furoate or fluticasone propionate. This study was approved by the local ethics committee (Comité Ético de Investigación Clínica–CEIm—reference HGUV 2/2016). All patients were given full information and the ones who consented to participate in this study were included. Computed tomography (CT) was compulsory before any surgical intervention and this was only considered in patients with no response to systemic steroid treatment or with complications as per the EPOS guidelines [1].

### 2.2. Exclusion Criteria

Patients with an autoimmune disease, children, unilateral polyposis, cystic fibrosis, primary ciliary dyskinesia and the presence of an upper airway infection 4 weeks prior to oral corticosteroid (CS) treatment were excluded from the study.

### 2.3. Patient Outcome Measures

Patients were given the quality of life (QoL) questionnaire the Sino-Nasal Outcome Test 22 (SNOT-22) after the end of the recruitment period and were asked to complete it.

### 2.4. Clinical and Molecular Parameters

The diagnosis of aspirin intolerance was established when patients reported a clinical reaction of nasal obstruction/rhinorrhea/dyspnea/skin rash when taking an NSAID and in doubtful cases by nasal provocation with lysine aspirin (LAS) [1,7]. Asthma was evaluated by a pneumologist and the diagnosis was made following the diagnostic criteria by the Spanish guidelines for asthma management (GEMA 4.3) [8]. Allergies were evaluated by an allergologist after performing skin prick testing or analyzing serum specific IgE for common inhaled allergens.

Pre-interventional demographic criteria were obtained from both the patient and their medical record as described in Table S1. The endoscopic polyposis score (EPS) was described as per Gevaert et al. [9] and the radiologic score (RS) was defined as per the Lund–Mackay computed tomography scoring [10,11]. Molecular parameters were defined as per previous studies from our group [12,13] and are shown in Table S2. MUC1 can modulate the CS response and could have a role in QoL [13]. CS response is influenced by MKP1, MIF and GRα. TLR2, TLR4 and TLR5 play pivotal roles in the innate immune system [14]. IL-8 mediates the granulocytic inflammatory response; it is a potent activating and chemotactic factor of neutrophils. It participates in the pathogenesis of a variety of neutrophil-infiltrating chronic inflammatory diseases [15,16].

### 2.5. Population Phenotypes

Medical management consisted of oral deflazacort 1 mg/kg/day for 8 days, then 0.5/mg/day for 7 days. Corticoresistance (CR) was defined as less than one degree of improvement in an endoscopic examination after the steroid treatment. The degree of corticosteroid response was identified as (0) no response, (1) clinical control with no endoscopic response, (2) clinical and endoscopic response < 3 months, (3) clinical and endoscopic response 3–6 months and (4) clinical and endoscopic response > 6 months.

Patients were classified considering phenotypic characteristics such as nasal polyposis (NP) without asthma and without aspirin intolerance (NPsA), NP with asthma and with aspirin tolerance (NP-AAT), NP with asthma and with aspirin intolerance (NP-AAI), NP without asthma and without aspirin intolerance corticosteroid resistant (NPsA-CR), NP with asthma and with aspirin tolerance corticosteroid resistant (NP-AAT-CR) and NP with asthma and with aspirin intolerance corticosteroid resistant (NP-AAI-CR). Corticoresistance was defined as mentioned above.

### 2.6. Quality of Life Evaluation

Consenting patients were asked to complete the specific QoL instrument SNOT-22 after completing treatment. The overall punctuation from the 22 items was calculated and the 5 symptoms that each patient indicated as the worst were recorded. The principal investigator was blind to all QoL responses for the study duration.

### 2.7. Sample Collection

Two weeks after stopping topical steroids, NP tissues were sampled in the clinic under a topical local anesthesia with lidocaine 2%, before starting oral corticosteroid therapy. The samples were preserved in formaldehyde 4% for at least 48 h (for the histological analysis) or RNAlater (for molecular studies).

### 2.8. Histological Evaluation

The specimens fixed in formaldehyde were afterwards dehydrated and included in paraffin blocks [12] by an inclusion EC350-1 Myr (Leica Geosystems, Heerbrugg, Switzerland) and then cut into 4–6 µm slices with a microtome HM 340 E (Leica Geosystems, Heerbrugg, Switzerland) [12]. After the paraffin was removed and the preparation was introduced into several xylene baths, the sample was washed with alcohol and finally with water, after which it was stained with hematoxylin–eosin. The eosinophil percentage was determined by the cellular count in four representative samples for each individual using an optical microscope (Eclipse E200; Nikon, Inc., Tokyo, Japan) with a graduated reticle mounted onto a digital camera (Coolpix 4500; Nikon, Inc., Tokyo, Japan) and photos were taken of selected fields at 100× and 400× (Coolpix 4500; Nikon, Inc., Tokyo, Japan). The eosinophil percentage was expressed in relation to inflammatory cells, excluding epithelial cells.

### 2.9. Molecular Evaluation

Ribonucleic acid (RNA) was isolated by cellular lysis. Total RNA was isolated by the extraction system ABI PrismTM 6100 Nucleic Acid Prep Station (Applied Biosystems, Darmstadt, Germany) and its concentration (ng/µL) was determined by the NanoDrop 2000C spectrophotometer (ThermoFisher Scientific, Waltham, MA, USA). The total RNA concentration (ng/µL) was determined by absorbance measures at 260 nm (A260) and 280 nm (A280). The purity sample value was determined by the ratio A260/A280, considering an absolute value between 1.7 and 2.1 to be accepted as a valid sample for the analysis. Only samples with adequate purity values were used for the genic expression analysis. RNA integrity was confirmed by the capillary electrophoresis system 2100 Bioanalyzer (Agilent Technologies, Palo Alto, CA, USA). Only samples with ribosomal ARN integrity were analyzed. The extracted RNA was stored at −80 °C.

Retrotranscription was performed for a total of 300 ng of RNA to complementary deoxyribonucleic acid (cDNA) using a “Taq Man retrotranscription Kit”, in a 9800 Fast Thermal Cycler (Applied Biosystems, Perkin-Elmer Corporation, CA, USA), following these steps: incubation at 25 °C for 10 min, 30 min cycle at 42 °C and enzyme inactivation at 95 °C for 5 min. The synthesized DNA was stored at −20 °C.

The real time or quantitative polymerase chain reaction (cPCR) was obtained by the gene expression assays TaqMan^®^ reverse transcription reagents kit. The cDNA was amplified with specific primers and probes predesigned for MUC1 (Hs00159357_m1), MKP1 (Hs00610256_g1), MIF (Hs00236988_g1), GRα (Hs00353740_m1), IL8 (Hs00174103_m1), TLR2 (Hs00610101_m1), TLR4 (Hs00152939_m1) and TLR5 (Hs01019558_m1) [13]. It was developed in the thermocycle 7900 HT Fast Real-Time PCR System (Applied Biosystems) using the following steps: a cycle of 2 min at 0 °C, one cycle of 10 min at 95 °C and 40 cycles of 15 s at 95 °C, followed by a cycle of 1 min at 60 °C.

Expression of the target gene was expressed as the fold increase or decrease relative to the expression of glyceraldehyde 3-phosphate dehydrogenase (GAPDH) as an endogenous control (Applied Biosystems; Cat number 4352339E). The mean value of the replicates for each sample was calculated and expressed as the cycle threshold (Ct). The level of gene expression was then calculated as the difference (ΔCt) between the Ct value of the target gene and the Ct value of GAPDH. The fold changes in the target gene mRNA levels were designated as 2 elevated to −ΔCt.

### 2.10. Statistical Analysis

All the clinical and molecular data as well as the QoL responses were recorded on standardized clinical research forms. The quantitative variables were defined by mean ± standard deviation and median (first and third quartiles). The absolute and relative frequencies were used for the categorical variables. Ordinal regression models were estimated in order to calculate the association between QoL and clinical and morphologic characteristics. Confidence intervals of 95% were obtained for all the parameters. Analysis was performed using the whole cohort of patients and not within the six subgroups, considering the small number of patients that existed in each subgroup.

A statistical analysis of the results was carried out with statistics software R (3.4.2 version) and ordinal data [2019.4-25 version (Christensen, R.H.B., 2019. Ordinal–Regression Models for Ordinal Data. R package version 2019.4-25), (R Core Team, 2017.) R: A language and environment for statistical computing. R Foundation for Statistical Computing, Vienna, Austria. URL: https://www.R-project.org (accessed on 27 February 2020)].

## 3. Results

A total of 62 patients met the inclusion criteria. The patients’ demographics, eosinophil degree, molecular characteristics and SNOT-22 outcomes are defined in the Supplementary Tables S1 and S2 considering the six previously defined phenotypic groups. The mean age was 55.4 years, 54.8% of the patients were women, 38.7% presented with allergies, 70.8% had asthma and 56.5 had an NSAID intolerance. In total, 15% of the patients were smokers. The EPS was 2.97 and 2.82 in the right and left nasal nostril, respectively, and the RS was 16. The mean surgical procedures was 1.17. The most commonly used corticosteroids were Mometasone Fuorate (MS) among the INCS and prednisone (P) out of the systemic corticosteroids. The mean eosinophil level was 46% being the highest concentration among NP-AAI, NP-AAI-CR, NP-AAT and NPsA. The mean MUC1 level was 1.02 with the lowest concentration found in the NP-AAI-CR and NPsA-CR groups.

### 3.1. SNOT-22

Results from the SNOT-22 questionnaire were obtained from 42 patients among the 62 included initially (Table 1). Among the 20 patients who did not complete the questionnaire, 9 did not finally complete the questionnaire after accepting to participate and signing the consent form, and 11 could not be contacted for this part of the study. The worst symptoms described by the patients included sense of taste/smell (69.1%), blockage/congestion of nose (64.3%), runny nose (42.9%), ear pain/pressure (28.6), fatigue during the day (26.2%) and need to blow nose (26.2%). The mean SNOT-22 punctuation was 39.8 with a standard deviation of 28.0 and a median of 35. Higher values corresponded to the NP-AAI and NP-AAI-CR groups (Supplementary Table S1).

### 3.2. Association between QoL and Clinical Parameters

A higher SNOT-22 punctuation, meaning worse QoL, correlated with a higher EPS LNN (*p* = 0.04) although not with an EPS RNN (*p* = 0.09) as seen in Table 2.

### 3.3. Association between QoL and Molecular Parameters

**Figure 1:**
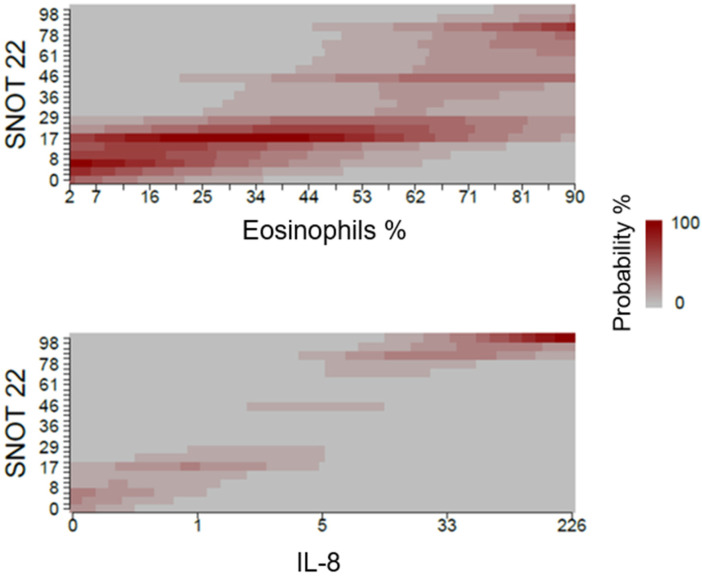
Association between SNOT-22 and the molecular parameters eosinophils and IL-8. The expression of target genes was expressed as 2 elevated—deltaCt.

QoL was correlated with IL-8 and with eosinophilia. Hence, patients with increased levels of IL-8 and patients with a higher eosinophil percentage had worse QoL (Table 3). As described in Figure 1, higher values of IL-8 expression were related to higher SNOT-22 scores. In the same way, a higher eosinophil percentage was associated with higher SNOT-22 values.

### 3.4. Association between QoL and the Parameters: Eosinophil Percentage, MUC1 Expression, NSAID Intolerance and Asthma

Considering all the variables mentioned above, high eosinophil levels were again correlated with worse QoL (*p* = 0.01). Moreover, in this case, an association between high SNOT-22 values and NSAID intolerance was also found (*p* = 0.04) (Table 4).

**Figure 2:**
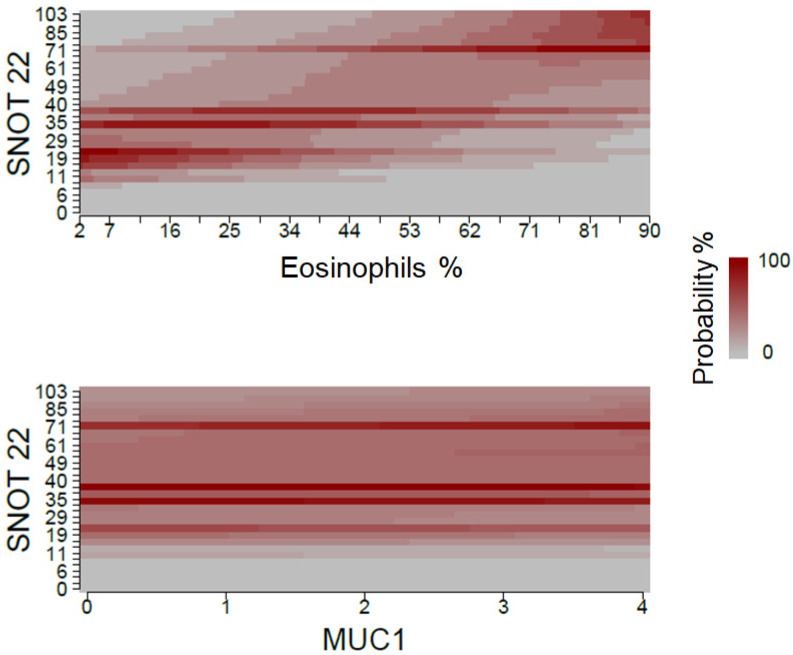
Association between eosinophil percentage and MUC1 expression, and QoL measured with SNOT-22. The expression of target genes was expressed as 2 elevated—deltaCt.

Figure 2 shows the fact that high eosinophil percentage values are associated with a greater probability of high SNOT-22 scores. On the other hand, the probability for each of MUC1 values is similar when considering different SNOT-22 results.

**Figure 3:**
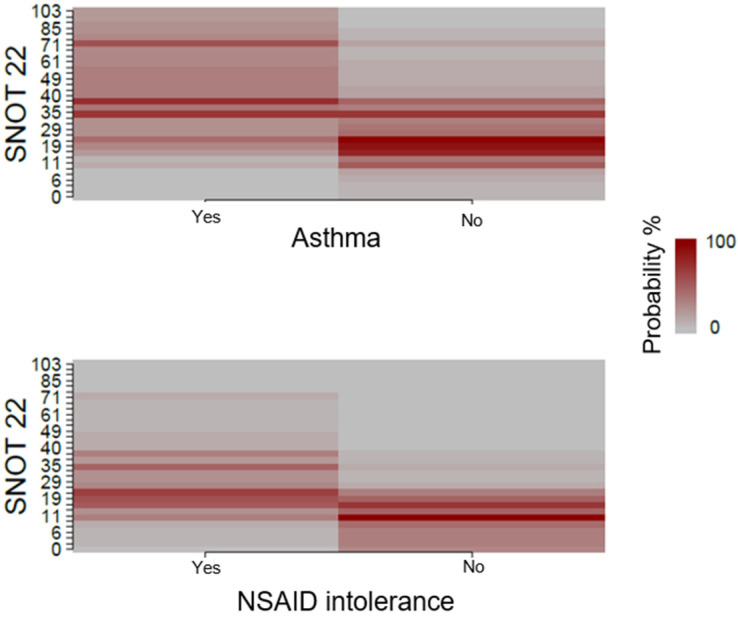
Association between asthma and NSAID intolerance, and QoL measured with SNOT-22.

Patients without an NSAID intolerance had a larger probability to have lower SNOT-22 values. Contrastingly, SNOT-22 values were higher for patients with an NSAID intolerance, as described in Figure 3. Asthma was not correlated with SNOT-22 values.

## 4. Discussion

Although different QoL questionnaires have been defined in patients with rhinologic disorders, including RSDI, CSS and SNOT-22, the EPOS recommends SNOT-22 as the QoL measurement tool in patients with CRSwNP. The SNOT-22 questionnaire, which was validated by Hopkins [5], is the most effective instrument to evaluate severity and QoL impact in patients’ clinical symptoms. In 2004, Kountakis [17] used the SNOT-20 tool to investigate the relation between the endoscopic and radiologic degree of polyposis and preoperative and postoperative QoL. They did not find any statistical association between them. Soler [18,19] and Smith [19] used the RSDI, CSS and SF-36 questionnaires in order to assess the QoL in patients with CRS. The mean SNOT-22 punctuation in the previous published literature ranges between 23 and 35–45%, being the lower range values described in a study from the Danish population [4,6,20]. In our study, it was 39.8.

Our cohort demonstrated that patients with a higher EPS LNN had significantly higher SNOT-22 scores, although this was not the case for EPS RNN. However, this statistical difference between LNN and RNN is scarce (*p* LNN = 0.04 versus *p* RNN = 0.09) and we consider that it has no clinical significance. As CRSwNP is considered a sinonasal disease involving the nasal mucosa as a whole, these results should be cautiously considered as one would expect that both nasal nostrils should behave in the same way in relation to QoL. A similar correlation was found by Mace [21] although using the RSDI and CSS system scores. In this case, the EPS 12 months after sinonasal surgery correlated with an improvement on the total RSDI score, the physical and functional subscales of the RSDI and the symptom subscale of the CSS, but could explain only 25.5–36.6% of the linear variation for these health-related quality of life improvements. Our impression is that further studies with a larger number of patients could confirm if the EPS in both nasal nostrils correlates with the SNOT-22.

Eosinophilic damage to the sinonasal mucosa was accepted to be the pathophysiologic mechanism of CRS and the hallmark of the disorder [1,22]. It was also demonstrated that tissue eosinophilia was higher in patients with CRSwNP than in patients with CRSsNP in western and Caucasian populations. Hence, eosinophils may be critical to polyp formation but they are not absolutely necessary to be present [23,24]. Eosinophils are a marker of severe and recalcitrant disease [1,25,26,27]. There is no established criterion to define eosinophilia. Tissue eosinophilia can be measured either in quantitative terms (number of eosinophils per field) or by percentages (with respect to the total number of inflammatory cells in the infiltrate) [28]. In Western countries, mucosal eosinophilia is defined as >5 or >10 eosinophils per high-power field (HPF) [25]. While some authors define it as more than 5% of eosinophils per high-power field (HPF) [17,18,29,30], others define it as more than 10% of eosinophils per HPF [19,27,31,32,33,34,35,36,37]. It has also been classified in different degrees (without eosinophilia ≤ 5%, minor eosinophilia ≤ 10%, moderate eosinophilia ≤ 50% or marked eosinophilia ≥ 50%), as it is the case of this study where eosinophilia was expressed in percentages [38,39]. In Japan, mucosal eosinophilia is defined as ≥70, >100 or >200 eosinophils per HPF [25,40,41,42].

There are limited studies investigating the role of eosinophils in QoL in patients with CRSwNP [18,19,26,27,31,33,36,43]. These previous studies used generic and specific questionnaires such as the Short-Form 36 (SF-36) [18,19,31], the Rhinosinusitis Disability Index (RSDI) [18,19,31,33], the Chronic Sinusitis Survey (CSS) [18,19,31], the Smell Identification Test (SIT) [36], the Patient-Reported Outcomes Measurement Information System-29 (PROMIS-29) [43], the SNOT-20 and the SNOT-22 test [20,26,27,43]. The results of our study reveal a significant association between higher eosinophil levels and a higher SNOT-22 punctuation. However, Soler [18,19] did not find any significant association between eosinophilia and QoL using the RSDI, CSS and SF-36 questionnaires in either of the two studies they published. Soy [33] also analyzed the impact of eosinophilia in patients’ QoL and they failed to correlate eosinophilia with RSDI, although it was correlated with a better improvement in the functional subscale of the SF-36. Likewise, no significant improvement in the RSDI and CSS questionnaires was found after surgery depending on eosinophil count in the work published by Smith [31]. Hauser [36] described significantly lower SIT scores in patients with tissular eosinophilia. Baudoin [44] found that eosinophilia was correlated with nasal secretion although they analyzed different symptoms but did not use a validated questionnaire. Using the SNOT-20 test, Kountakis did not report any relation between eosinophilia and subjective symptom scores [17]. Among the authors that used the SNOT-22 questionnaire in patients with CRSwNP, none of them reported a statistically significant association between eosinophilia and SNOT-22 scores [26,27,32,43]. One study reported a correlation between tissue eosinophilia and SNOT-22 scores but in patients with CRSsNP [20]. Our study showed a similar relationship in patients with CRSwNP. Hence, patients with a high eosinophil percentage demonstrated significantly higher SNOT-22 scores. To our knowledge, our study presents the first study correlating the degree of eosinophilia with worse QoL measured by SNOT-22.

Considering the different groups in our cohort, the higher number of eosinophils was found in the groups NP-AAI, NP-AAI-CR, NP-AAT and NPsA. One could understand that patients with asthma, aspirin intolerance or CR could present a higher eosinophil count, but we also found high eosinophil counts in patients with NPsA. Nonetheless, these are descriptive results and no statistical analysis was performed as per the small number of patients included in each group.

IL-8 is a proinflammatory cytokine and it is a potent neutrophil-activating and chemotactic factor. It participates in the pathogenesis of various neutrophil-infiltrating chronic inflammatory diseases, suggesting a reciprocal relationship between neutrophils and IL-8 in CRS [15]. Neutrophils are capable of producing IL-8 in response to several kinds of stimuli. This induces further neutrophil accumulation in the sinus effusion of patients with chronic sinusitis [15]. Therefore, IL-8 is considered a neutrophilic inflammatory marker [45]. Huriyati defined that IL-8 expression in nasal polyp tissue was significantly lower in recurrent CRSwNP than in non-recurrent CRSwNP [3]. Wei also concluded that IL-8 values were higher in the non-recurrent group although its positivity was not significantly different between recurrent and non-recurrent CRSwNP [45]. A significant reduction in IL-8 expression after treatment with INCS and clarithromycin has been found. Similarly, patients with a high IL-8 concentration prior to treatment had a better response [46]. In contrast, another study did not find a correlation between IL-8 levels and recurrence after surgery [47]. These studies did not study the effect of IL-8 in patients’ QoL. Our cohort demonstrated that high IL-8 levels were correlated with higher SNOT-22 scores. This is, to our knowledge, the first study that has found a significant association between these two parameters, providing new evidence about QoL markers in patients with CRSwNP. This could be in line with the previous published studies that described a positive correlation between IL-8 and response to treatment.

NSAID hypersensitivity is closely associated with concomitant respiratory disease [4]. We have demonstrated that NSAID intolerance correlates with worse QoL measured with the SNOT-22 tool when also considering the influence of MUC1, asthma and eosinophils. Nonetheless, this association was not significant when analyzing NSAID intolerance along with the other clinical variables. Previous published research described a higher prevalence of NSAID intolerance in relation to higher SNOT-22 values although this association was not significant [4]. In a tertiary referral centre, the prevalence of NSAID-exacerbated respiratory disease (N-ERD) among patients with CRSwNP was 16% [48]. Patients with N-ERD significantly underwent two-fold more sinus surgeries and were significantly younger at the time of their first surgery than patients with CRSwNP without N-ERD [1,48]. It has not previously been demonstrated that NSAID hypersensitivity is correlated with SNOT-22 outcomes. Overall, this should cautiously be considered as per the controversy of our results having only found this correlation in one of the two analyses mentioned above.

Mucins are the major components of the airway epithelial mucus layer that contribute to the protection of the epithelium from pathogens and irritants, having a role in the mucociliary clearance mechanism [49,50]. MUC1 in relation to QoL has not been investigated in the past. Our study failed to demonstrate an association between MUC1 expression and QoL. In the descriptive analyses, we established that MUC1 was lower in patients with CR (NP-AAI-CR and NPsA-CR). Although no statistical analyses were performed considering the small number of patients included in each group, this was in line with the previous results from our group [13].

Value of the study: Up until now no studies have summarized the association between molecular markers and the SNOT-22 questionnaire. This is the first study that describes a correlation between the SNOT-22 and IL-8 expression, eosinophil percentage and NSAID intolerance. This should aid in predicting the quality of life in patients with CRSwNP by considering these markers.

Limitations of the study: The limitations of this study are the number of cases included in the analysis. Considering it was an exploratory and ambispective observational study, the sample size could not be calculated, so in this study we have included all our patients. Focusing specifically on some variables, it may account for the controversy in significant results in only one of the two nasal nostrils for the EPS and to the fact that NSAID intolerance has significantly been associated with worse QoL considering eosinophilia, asthma and the SNOT-22 but not when considering all the other clinical parameters. Moreover, the SNOT-22 was circulated at the same period of time for all the patients regardless of when their treatment was completed. Further studies designing a specific time before and after treatment might be useful in the future.

## 5. Conclusions

Eosinophilia, IL-8 expression and NSAID intolerance can be used as predictors of QoL in patients with CRSwNP. Knowing the predictor factors of a patient’s QoL in CRSwNP will aid a better understanding of this disease and to improve a patient’s management.

## Figures and Tables

**Table 1:** Percentage of patients who highlighted these items of the questionnaire as being one of the 5 worst symptoms.

Symptom	%
1. Need to blow nose	26.19
2. Sneezing	19.05
3. Runny nose	42.86
4. Cough	9.52
5. Post nasal discharge	9.52
6. Thick nasal discharge	21.43
7. Ear fullness	4.76
8. Dizziness	4.76
9. Ear pain/pressure	28.57
10. Facial pain/pressure	14.29
11. Difficulty falling asleep	4.76
12. Waking up at night	9.52
13. Lack of a good night’s sleep	16.67
14. Waking up tired	14.29
15. Fatigue during the day	26.19
16. Reduced productivity	2.38
17. Reduced concentration	4.76
18. Frustrated/restless/irritable	14.29
19. Sad	9.52
20. Embarrassed	4.76
21. Sense of taste/smell	69.05
22. Blockage/congestion of nose	64.29

**Table 2:** Association between QoL measured by SNOT-22 and clinical parameters. RS: radiologic scoring, EPS LNN: endoscopic polyposis score left nasal nostril, EPS RNN: endoscopic polyposis score right nasal nostril, IN CC: intranasal corticosteroids, NSAID: intolerance non-steroidal anti-inflammatory drug intolerance.

	Std. Error	Odds Ratio	Lower 95%	Upper 95%	*p* Value
RS	0.07	1.09	0.96	1.25	0.19
EPS LNN	0.43	0.4	0.17	0.93	0.04
EPS RNN	0.55	2.54	0.88	7.70	0.09
IN CC	0.27	1.46	0.867	2.49	0.16
Systemic corticosteroids	0.35	0.64	0.32	1.24	0.20
Allergy	0.69	0.93	0.24	3.58	0.92
Smoker	0.82	1.21	0.24	6.22	0.81
Asthma	1.28	9.65	0.86	134.32	0.08
NSAID intolerance	1.03	2.9	0.40	24.60	0.30
Age	0.03	0.98	0.93	1.04	0.54
Sex	0.77	0.90	0.20	4.18	0.90

**Table 3:** Association between SNOT-22 and molecular parameters. IL-8 interleukin 8, MUC1 mucin 1, MKP1 mitogen-activated protein kinase phosphatase 1, MIF macrophage migration inhibitory factor, GRα Glucocorticoid receptor α, TLR2 toll-like receptor 2, TLR4 toll-like receptor 4, TLR5 toll-like receptor 5.

	Std. Error	Odds Ratio	Lower 95%	Upper 95%	*p* Value
IL-8_log	0.57	4.90	1.64	16.76	0.01
MUC1	0.91	1.79	0.20	11.31	0.52
MKP1	0.91	2.81	0.43	17.62	0.26
MIF	2.80	0.01	0	2.58	0.12
GRα	1.11	0.17	0.02	1.38	0.11
TLR2	1.13	0.16	0.02	1.40	0.10
TLR4_log	0.63	0.49	0.09	1.22	0.15
TLR5	1.88	4.29	0.12	215.58	0.44
Eosinophils (%)	0.023	1.06	1.01	1.12	0.02

**Table 4:** Association between SNOT-22 and the parameters Eosinophil percentage, MUC1 expression, NSAID intolerance and asthma. MUC1 mucin 1, NSAID non-steroidal drugs intolerance.

	Std. Error	Odds Ratio	Lower 95%	Upper 95%	*p* Value
Eosinophils (%)	0.01	1.03	1.01	1.06	0.01
MUC1	0.33	1.07	0.54	2.06	0.83
Asthma	0.99	5.31	0.79	41.27	0.09
NSAID intolerance	0.96	7.09	1.18	51.99	0.04

## Data Availability

Not applicable.

## Supplementary Materials

The following supporting information can be downloaded at: https://www.mdpi.com/article/10.3390/jcm12041391/s1. Table S1: Descriptive clinical data; Table S2: Descriptive molecular data.

## References

[1] Fokkens W.J., Lund V.J., Hopkins C., Hellings P.W., Kern R., Reitsma S., Toppila-Salmi S., Bernal-Sprekelsen M., Mullol J., Alobid I. (2020). European position paper on rhinosinusitis and nasal polyps EPOS 2020. Rhinology.

[2] Abdalla S., Alreefy H., Hopkins C. (2012). Prevalence of Sinonasal Outcome Test (SNOT-22) symptoms in patients undergoing surgery for Chronic Rhinosinusitis. Clin. Otolaryngol..

[3] Huriyati E., Darwin E., Yanwirasti Y., Wahid I. (2019). Differences in expression of inflammatory mediator in mucosal and polyp tissue between chronic rhinosinusitis and recurrent chronic rhinosinusitis. Maced. J. Med. Sci..

[4] Lange B., Mortz C.G., Bindslev-Jensen C., Kjeldsen A.D. (2019). Nasal symptoms in patients with NSAID hypersensitivity. Rhinol. Online.

[5] Hopkins C., Gillett S., Slack R., Lund V.J., Browne J.P. (2009). Psychometric validity of the 22-item Sinonasal Outcome Test. Clin. Otolaryngol..

[6] Browne J.P., Hopkins C., Slack R., Cano S.J. (2007). The Sino-Nasal Outcome Test (SNOT): Can we make it more clinically meaningful?. Otolaryngol. Head Neck Surg..

[7] Wöhrl S. (2018). NSAID hypersensitivity—Recommendations for diagnostic work up and patient management. Allergo J. Int..

[8] GEMA 4.3 (2018). Guia Española Para el Manejo del Asma.

[9] Gevaert P., Calus L., Van Zele T., Blomme K., De Ruyck N., Bauters W., Hellings P., Brusselle G., De Bacquer D., Van Cauwenberge P. (2013). Omalizumab is effective in allergic and nonallergic patients with nasal polyps and asthma. J. Allergy Clin. Immunol..

[10] Lund V.J., Mackay I.S. (1993). Staging rhinosinusitis. Lund 1993.pdf. Rhinology.

[11] Lund V.J., Kennedy D.W. (1997). Staging for rhinosinusitis. Otolaryngol. Head Neck Surg..

[12] Milara J., Morell A., Ballester B., Armengot M., Morcillo E., Cortijo J. (2016). MUC4 impairs the anti-inflammatory effects of corticosteroids in patients with chronic rhinosinusitis with nasal polyps. J. Allergy Clin. Immunol..

[13] Milara J., Peiró T., Armengot M., Frias S., Morell A., Serrano A., Cortijo J. (2015). Mucin 1 downregulation associates with corticosteroid resistance in chronic rhinosinusitis with nasal polyps. J. Allergy Clin. Immunol..

[14] Kawasaki T., Kawai T. (2014). Toll-like receptor signaling pathways. Front. Immunol..

[15] Suzuki H., Takahashi Y., Wataya H., Ikeda K., Nakabayashi S., Shimomura A., Takasaka T. (1996). Mechanism of neutrophil recruitment induced by IL-8 in chronic sinusitis. J. Allergy Clin. Immunol..

[16] Wang Y., Li C., Liu H. (2021). [The expression and role of interleukin-8 in chronic rhinosinusitis]. Lin Chung Er Bi Yan Hou Tou Jing Wai Ke Za Zhi.

[17] Kountakis S.E., Arango P., Bradley D., Wade Z.K., Borish L. (2004). Molecular and Cellular Staging for the Severity of Chronic Rhinosinusitis. Laryngoscope.

[18] Soler Z.M., Sauer D.A., Mace J., Smith T.L. (2009). Relationship between clinical measures and histopathologic findings in chronic rhinosinusitis. Otolaryngol. Head Neck Surg..

[19] Soler Z.M., Sauer D.A., Mace J., Smith T.L. (2010). Impact of Mucosal Eosinophilia and Nasal Polyposis on Quality of Life Outcomes after Sinus Surgery. Otolaryngol. Head Neck Surg..

[20] Lal D., Hopkins C., Divekar R.D. (2018). SNOT-22-based clusters in chronic rhinosinusitis without nasal polyposis exhibit distinct endotypic and prognostic differences. Int. Forum Allergy Rhinol..

[21] Mace J.C., Michael Y.L., Carlson N.E., Litvack J.R., Smith T. (2010). Correlations Between Endoscopy Score and Quality-of-Life Changes After Sinus Surgery. Arch Otolaryngol. Head Neck Surg..

[22] Harlin S.L., Ansel D.G., Lane S.R., Myers J., Kephart G.M., Gleich G.J. (1988). A clinical and pathologic study of chronic sinusitis: The role of the eosinophil. J. Allergy Clin. Immunol..

[23] Payne S.C., Early B., Huyett P., Han J.K., Borish L., Steinke J.W. (2011). Evidence for distinct histological profile of nasal polyps: With and without eosinophilia. Laryngoscope.

[24] Zhang Y., Gevaert E., Lou H., Wang X., Zhang L., Bachert C., Zhang N. (2017). Chronic rhinosinusitis in Asia. J. Allergy Clin. Immunol..

[25] Tokunaga T., Sakashita M., Haruna T., Asaka D., Takeno S., Ikeda H., Nakayama T., Seki N., Ito S., Murata J. (2015). Novel scoring system and algorithm for classifying chronic rhinosinusitis: The JESREC Study. Allergy Eur. J. Allergy Clin. Immunol..

[26] Tajudeen B.A., Ganti A., Kuhar H.N., Mahdavinia M., Heilingoetter A., Gattuso P., Ghai R., Batra P.S. (2019). The presence of eosinophil aggregates correlates with increased postoperative prednisone requirement. Laryngoscope.

[27] Kuhar H.N., Tajudeen B.A., Mahdavinia M., Gattuso P., Ghai R., Batra P.S. (2017). Inflammatory infiltrate and mucosal remodeling in chronic rhinosinusitis with and without polyps: Structured histopathologic analysis. Int. Forum Allergy Rhinol..

[28] Garín L., Armengot M., Alba R., Carda C. (2008). Correlations Between Clinical and Histological Aspects in Nasal Polyposis. Acta Otorrinolaringol. Esp..

[29] Armengot M., Garín L., De Lamo M., Krause F., Carda C. (2010). Cytological and tissue eosinophilia correlations in nasal polyposis. Am. J. Rhinol. Allergy.

[30] Vlaminck S., Vauterin T., Hellings P.W., Jorissen M., Acke F., Van Cauwenberge P., Bachert C., Gevaert P. (2014). The importance of local eosinophilia in the surgical outcome of chronic rhinosinusitis: A 3-year prospective observational study. Am. J. Rhinol. Allergy.

[31] Smith T.L., Litvack J.R., Hwang P.H., Todd A., Mace J.C., Fong K.J., James K.E. (2010). Determinants of Outcomes of Sinus Surgery: A Multi-Institutional Prospective Cohort Study. Otolaryngol. Head Neck Surg..

[32] Snidvongs K., Lam M., Sacks R., Earls P., Kalish L., Phillips P.S., Pratt E., Harvey R.J. (2012). Structured histopathology profiling of chronic rhinosinusitis in routine practice. Int. Forum Allergy Rhinol..

[33] Soy F.K., Pinar E., Imre A., Calli C., Calli A., Oncel S. (2013). Histopathologic parameters in chronic rhinosinusitis with nasal polyposis: Impact on quality of life outcomes. Int. Forum Allergy Rhinol..

[34] Gitomer S.A., Fountain C.R., Kingdom T.T., Getz A.E., Sillau S.H., Katial R.K., Ramakrishnan V.R. (2016). Clinical Examination of Tissue Eosinophilia in Patients with Chronic Rhinosinusitis and Nasal Polyposis. Otolaryngol. Head Neck Surg..

[35] Sreeparvathi A., Kalyanikuttyamma L.K., Kumar M., Sreekumar N., Veerasigamani N. (2017). Significance of blood eosinophil count in patients with chronic rhinosinusitis with nasal polyposis. J. Clin. Diagn. Res..

[36] Hauser L.J., Chandra R.K., Li P., Turner J.H. (2017). Role of tissue eosinophils in chronic rhinosinusitis-associated olfactory loss. Int. Forum Allergy Rhinol..

[37] Aslan F., Altun E., Paksoy S., Turan G. (2017). Could Eosinophilia predict clinical severity in nasal polyps?. Multidiscip. Respir. Med..

[38] Armengot M., Garín L., Carda C. (2009). Eosinophil degranulation patterns in nasal polyposis: An ultrastructural study. Am. J. Rhinol. Allergy.

[39] Mygind N. (1982). Alergia Nasal: Leucotios Eosinófilos.

[40] Nakayama T., Yoshikawa M., Asaka D., Okushi T., Matsuwaki Y., Otori N., Hama T., Moriyama H. (2011). Mucosal eosinophilia and recurrence of nasal polyps—New classification of chronic rhinosinusitis. Rhinology.

[41] Matsuwaki Y., Ookushi T., Asaka D., Mori E., Nakajima T., Yoshida T., Kojima J., Chiba S., Ootori N., Moriyama H. (2008). Chronic Rhinosinusitis: Risk Factors for the Recurrence of Chronic Rhinosinusitis Based on 5-Year Follow-Up after Endoscopic Sinus Surgery. Int. Arch. Allergy Immunol..

[42] Ikeda K., Shiozawa A., Ono N., Kusunoki T., Hirotsu M., Homma H., Saitoh T., Murata J. (2013). Subclassification of chronic rhinosinusitis with nasal polyp based on eosinophil and neutrophil. Laryngoscope.

[43] Thompson C.F., Price C.P.E., Huang J.H., Min J.Y., Suh L.A., Shintani-Smith S., Conley D.B., Schleimer R.P., Kern R.C., Tan B.K. (2016). A pilot study of symptom profiles from a polyp vs an eosinophilic-based classification of chronic rhinosinusitis. Int. Forum Allergy Rhinol..

[44] Baudoin T., Čupić H., Geber G., Vagić D., Grgić M., Kalogjera L. (2006). Histopathologic parameters as predictors of response to endoscopic sinus surgery in nonallergic patients with chronic rhinosinusitis. Otolaryngol. Head Neck Surg..

[45] Wei B., Liu F., Zhang J., Liu Y., Du J., Liu S., Zhang N., Bachert C., Meng J. (2018). Multivariate analysis of inflammatory endotypes in recurrent nasal polyposis in a chinese population. Rhinology.

[46] Deng J., Chen F., Lai Y.Y., Luo Q., Xu R., Ou C., Fu Q., Shi J. (2018). Lack of additional effects of long-term, low-dose clarithromycin combined treatment compared with topical steroids alone for chronic rhinosinusitis in China: A randomized, controlled trial. Int. Forum Allergy Rhinol..

[47] Davide R., Chiara R., Giulio P., Bruna C., Carlo D.R., Claudio D.C., Salvatore M., Andrea G. (2020). Predictive markers of long-term recurrence in chronic rhinosinusitis with nasal polyps. Am. J. Otolaryngol. Head Neck Med. Surg..

[48] Stevens W.W., Peters A.T., Hirsch A.G., Nordberg C.M., Schwartz B.S., Mercer D.G., Mahdavinia M., Grammer L.C., Hulse K.E., Kern R.C. (2017). Clinical Characteristics of Patients with Chronic Rhinosinusitis with Nasal Polyps, Asthma, and Aspirin-Exacerbated Respiratory Disease. J. Allergy Clin. Immunol. Pract..

[49] Peiro T., Milara J., Armengot M., Cortijo J. (2014). Mucin Expression and Corticosteroid Efficacy in Chronic Rhinosinusitis with Nasal Polyps. J. Steroids Horm. Sci..

[50] Rose M.C., Voynow J.A. (2006). Respiratory tract mucin genes and mucin glycoproteins in health and disease. Physiol. Rev..

