# Supplementary material for: Molecular and Clinical Predictors of Quality of Life in Chronic Rhinosinusitis with Nasal Polyps
Source: J Clin Med. 2023 Feb 9;12(4):1391. doi: 10.3390/jcm12041391 (PMC9965377; doi:10.3390/jcm12041391)
Supplement: Supplementary file 1 [file jcm-12-01391-s001.zip › jcm-2078118-supplementary.pdf]

Table S1. Descriptive clinical data.

Var	NP-AAI	NP-AAI-CR	NP-AAT	NP-AAT-CR	NPsA	NPsA-CR	Total
	**n=27**	**n=8**	**n=7**	**n=2**	**n=11**	**n=7**	**n = 62**
	Mean(SD)/n(%)	Mean (SD)/n(%)	Mean (SD)/n(%)	Mean (DS)/n(%)	Mean (DS)/n(%)	Mean (DS)/n(%)	Mean (DS)/ n(%)
	Median (Q1,Q3)	Median (Q1,Q3)	Median (Q1,Q3)	Median (Q1,Q3)	Median (Q1,Q3)	Median (Q1,Q3)	Median (Q1,Q3)
**Age**							
	56.44 (13.7)	49.88 (15.9)	56.29 (8.6)	70.5 (12.0)	57.36 (11.8)	49.43 (14.7)	55.4 (13.4)
	56 (48, 67.5)	49 (35.5, 58.3)	55 (52, 61.5)	70.5(66.3, 74.8)	58 (49, 66.5)	52 (41.5, 6)	56 (45.8, 66)
**Sex**							
**F**	19 (70.4%)	6 (75%)	4 (57.1%)	1 (50%)	2 (18.2%)	2 (28.6%)	34 (54.8%)
**M**	8 (29.6%)	2 (25%)	3 (42.9%)	1 (50%)	9 (81.8%)	5 (71.4%)	28 (45.2%)
**Allergy**							
**No**	15 (55.6%)	4 (50%)	4 (57.1%)	1 (50%)	8 (72.7%)	6 (85.7%)	38 (61.3%)
**Yes**	12 (44.4%)	4 (50%)	3 (42.9%)	1 (50%)	3 (27.3%)	1 (14.3%)	24 (38.7%)
**Smoker**							
**No**	23 (85.2%)	8 (100%)	6 (85.7%)	2 (100%)	9 (81.8%)	5 (71.4%)	53 (85.5%)
**Yes**	4 (14.8%)	0 (0%)	1 (14.3%)	0 (0%)	2 (18.2%)	2 (28.6%)	9 (14.5%)

**Asthma**							
**No**	0 (0%)	0 (0%)	0 (0%)	0 (0%)	11 (100%)	7 (100%)	18 (29.0%)
**Yes**	27 (100%)	8 (100%)	7 (100%)	2 (100%)	0 (0%)	0 (0%)	44 (71.0%)
**NSAID intolerance**							
**No**	0 (0%)	0 (0%)	7 (100%)	2 (100%)	11 (100%)	7 (100%)	27 (43.6%)
**Yes**	27 (100%)	8 (100%)	0 (0%)	0 (0%)	0 (0%)	0 (0%)	35 (56.5%)
**EPS RNN**							
	2.96 (0.9)	3.17 (0.4)	2.71 (1.0)	3.5 (0.7)	2.91 (0.7)	3 (0.6)	2.97 (0.8)
	3 (2, 4)	3 (3, 3)	2 (2, 3.5)	3.5(3.3,3.8)	3 (2.5, 3)	3 (3, 3)	3 (2, 4)
**EPS LNN**							
	2.85 (1.1)	3.17 (0.41)	3 (0.8)	3.5 (0.7)	2.36 (1.0)	2.71 (1.0)	2.82 (1.0)
	3 (2, 4)	3 (3, 3)	3 (2.5, 3.5)	3.5 (3.3, 3.8)	2 (2, 3)	3 (2.5, 3)	3 (2, 4)
**RS**							
	17.6 (6.9)	18 (7.6)	13.83 (6.5)	16 (11.3)	13.36 (5.4)	13.6 (3.05)	15.96 (6.6)
	20 (12, 24)	21 (13.5, 24)	15 (12.5, 18.3)	16 (12, 20)	12 (10, 18)	13 (12, 15)	16 (10.5, 22)
**Number of surgical procedures**							
	1.42 (1.2)	2.17 (1.2)	0.86 (0.7)	0.5 (0.7)	0.55 (0.5)	0.86 (0.4)	1.17 (1.0)
	1 (1, 2)	2 (1.3, 2.8)	1 (0.5, 1)	0.5 (0.3, 0.8)	1 (0, 1)	1 (1, 1)	1 (1, 2)

**Response to INCS**							
	1.44 (1.5)	0.33 (0.5)	1 (1.5)	0.5 (0.7)	2.18 (1.7)	0.71 (1.1)	1.3 (1.5)
	1 (0, 2)	0 (0, 0.8)	0 (0, 1.5)	0.5 (0.3, 0.8)	2 (0.5, 4)	0 (0, 1)	1 (0, 2)
**Response to systemic corticosteroids**							
	3 (0.8)	0.33 (0.5)	3.33 (0.8)	0 (0)	3.5 (0.71)	0 (0)	2.38 (1.52)
	3 (2, 4)	0 (0, 0.75)	3.5 (3, 4)	0 (0, 0)	4 (3, 4)	0 (0, 0)	3 (1.25, 4)
**Corticosteroid resistance (CR)**							
**NO**	27 (100%)	0 (0%)	7 (100%)	0 (0%)	11 (100%)	0 (0%)	45 (72.58%)
**SI**	0 (0%)	8 (100%)	0 (0%)	2 (100%)	0 (0%)	7 (100%)	17 (27.42%)
**SNOT-22**							
	49.7 (22.8)	62.7 (51.2)	38.2 (26.1)	9.0	13.9 (14.5)	19.5 (16.3)	39.8 (28.0)
	40.5 (34.3, 65.8)	86.0 (45, 9)	34.5 (14.8, 58.8)	9.0 (9.0, 9.0)	10.0 (5.3, 17.5)	19.5 (13.8, 25.3)	35.0 (17.0, 60.5)

Var variable, NP-AAI Nasal polyps with asthma and aspirin intolerance, NP-AAI-CR Nasal polyps with asthma and aspirin intolerance corticoresistant, NP-AAT nasal polyps with asthma and aspirin tolerance, NP-AAT-CR nasal polyps with asthma and aspirin tolerance corticoresistant, NPsA nasal polyps without asthma, NPsA-CR nasal polyps without asthma corticoresistant, EPS RNN endoscopic polyposis score right nasal nostril, EPS LNN endoscopic polyposis score left nasal nostril, RS Radiologic scoring.

Table S2. Descriptive molecular data.

Var	NP-AAI<br>n=27<br>Mean(SD)/n(%)<br>Median (Q1,Q3)	NP-AAI-CR<br>n=8<br>Mean (SD)/n(%)<br>Median (Q1,Q3)	NP-AAT<br>n=7<br>Mean (SD)/n(%)<br>Median (Q1,Q3)	NP-AAT-CR<br>n=2<br>Mean (DS)/n(%)<br>Median (Q1,Q3)	NPsA<br>n=11<br>Mean (DS)/n(%)<br>Median (Q1,Q3)	NPsA-CR<br>n=7<br>Mean (DS)/n(%)<br>Median (Q1,Q3)	Total<br>n = 62<br>Mean (DS)/ n(%)<br>Median (Q1,Q3)
**Eosinophils (%)**							
	51.5 (24.2)	63.3 (20.6)	54.2 (30.7)	25 (7.1)	40.8 (31.7)	18.1 (23.5)	45.9 (28.0)
	50.0 (30.0, 71.3)	65.0 (45.0, 77.5)	65.0 (27.5, 8.0)	25.0 (22.5, 27.5)	40.0 (10.0, 67.5)	10.0 (7.5, 15)	50.0 (20.0, 7.0)
**MUC1**							
	1.2 (0.85)	0.29 (0.22)	1.08 (1.12)	1.0 (0.1)	1.14 (0.5)	0.8 (0.7)	1.02 (0.8)
	1.01 (0.7, 1.4)	0.25 (0.2, 0.4)	0.67 (0.4, 1.1)	1.0 (0.97, 1.0)	1.2 (0.8, 1.4)	0.56 (0.3, 1.1)	0.86 (0.5, 1.2)
**MKP1**							
	0.8 (0.5)	0.2 (0.2)	0.7 (0.4)	1.6 (2.1)	1.0 (0.4)	1.0 (1.1)	0.8 (0.7)
	0.6 (0.4, 1.0)	0.1 (0.0, 0.3)	0.7 (0.4, 1)	1.6 (0.9, 2.4)	1.0 (0.8, 1.2)	0.6 (0.2, 1.3)	0.7 (0.3, 1.1)
**MIF**							
	1.1 (1.0)	0.9 (0.2)	0.9 (0.3)	1.0 (0.1)	1.1 (0.9)	0.9 (0.5)	1.0 (0.8)
	0.9 (0.8, 1.0)	0.9 (0.8, 1.0)	0.9 (0.8,1.0)	1.0 (0.95, 1)	0.9 (0.7, 1.1)	0.9 (0.7, 1.0)	0.9 (0.8, 1.0)

Var	NP-AAI	NP-AAI-CR	NP-AAT	NP-AAT-CR	NPsA	NPsA-CR	Total
**GRα**							
	0.8 (0.5)	549 (0.4)	0.7 (0.7)	0.8 (0.4)	1.1 (0.67)	0.7 (0.31)	0.8 (0.6)
	0.7 (0.5, 1.0)	0.3 (0.3, 0.7)	0.6 (0.2, 0.8)	0.82 (0.7, 1.0)	0.9 (0.7, 1.2)	0.8 (0.5, 0.9)	0.71 (0.4, 1.0)
**IL-8**							
	6.7 (10.7)	73.1 (95.5)	1.4 (0.76)	4.52 (5.35)	1.46 (1.18)	1.51 (1)	11.1 (35.81)
	3.1 (0.7, 5.9)	17.1(10.1, 111.4)	1.2 (1.0, 1.3)	4.52 (2.6, 6.4)	1.19 (0.6, 2.3)	1.61 (1.11, 2.0)	1.43 (0.8, 4.2)
**TLR2**							
	1.2 (1.0)	4.59 (2.7)	0.9 (0.5)		1.1 (0.5)	1.1 (0.3)	1.8 (1.7)
	1 (0.6, 1.4)	4.7 (2.8, 6.4)	0.97 (0.6, 1.0)		1.0 (0.9, 1.2)	1.0 (0.9, 1.3)	1.0 (0.8, 1.4)
**TLR4**							
	1.6 (2.2)	17.2 (27.7)	0.7 (0.3)	3.1 (3.7)	1.32 (1.4)	1.1 (1.3)	2.59 (8.1)
	0.8 (0.34, 1.5)	4.1 (3.4, 17.8)	0.67 (0.5, 1)	3.1 (1.8, 4.4)	1.0 (0.5, 1.2)	0.5 (0.4, 1.1)	0.79 (0.4, 1.5)
**TLR5**							
	1.3 (1.3)	2.7 (2.4)	1.1 (0.7)	1.35 (0.5)	1.0 (0.4)	1.5 (0.8)	1.4 (1.3)
	0.7 (0.5, 1.8)	1.86 (1.2, 4)	1 (0.7, 1.3)	1.35 (1.2, 1.5)	1.1 (0.6, 1.3)	1.0 (1, 1.7)	1 (0.7, 1.5)

Var variable, NP-AAI Nasal polyps with asthma and aspirin intolerance, NP-AAI-CR Nasal polyps with asthma and aspirin intolerance corticoresistant, NP-AAT nasal polyps with asthma and aspirin tolerance, NP-AAT-CR nasal polyps with asthma and aspirin tolerance corticoresistant, NPsA nasal polyps

without asthma, NPsA-CR nasal polyps without asthma corticoresistant. MUC1 Mucin 1, MKP1 Mitogen-activated protein kinase, MIF Macrophage migration inhibitory factor, GRα Glucocorticoid receptor α, IL-8 Interleukin 8, TLR4 Toll-like receptor 4, TLR5 Toll-like receptor 5, TLR8, Toll-like receptor 8.
